# Surface Immobilization of pH-Responsive Polymer Brushes on Mesoporous Silica Nanoparticles by Enzyme Mimetic Catalytic ATRP for Controlled Cargo Release

**DOI:** 10.3390/polym8080277

**Published:** 2016-08-02

**Authors:** Hang Zhou, Xin Wang, Jun Tang, Ying-Wei Yang

**Affiliations:** College of Chemistry, International Joint Research Laboratory of Nano-Micro Architecture Chemistry (NMAC), Jilin University, Changchun 130012, China; zhouhang0126@126.com (H.Z.); xinwangjlu@163.com (X.W.)

**Keywords:** drug delivery, enzyme memetic, mesoporous silica nanoparticle, nanomedicine, polymer brush, SI-ATRP

## Abstract

Peroxidase mimetic catalytic atom transfer radical polymerization (ATRP) was first used to install tertiary amine-functionalized polymer brushes on the surface of mesoporous silica nanoparticles (MSNs) in a facile and highly efficient manner. Poly(*N*,*N*-dimethylaminoethyl methacrylate) (PDMAEMA) brushes-grafted MSNs were fabricated by biocompatible deuterohemin-β-Ala-His-Thr-Val-Glu-Lys (DhHP-6)-catalyzed surface-initiated ATRP (SI-ATRP). The resulting organic–inorganic hybrid nanocarriers were fully characterized by Fourier transform-infrared spectroscopy (FT-IR), thermogravimetric analysis (TGA), X-ray photoelectron spectroscopy (XPS), powder X-ray diffraction (XRD), SEM, TEM, Elemental analysis, Zeta-potential, and N_2_ adsorption–desorption isotherms, which demonstrated the successful coating of pH-responsive polymers on the MSN surface. Rhodamine 6G (Rh6G) dyes were further loaded within the mesopores of this nanocarrier, and the release of Rh6G out of MSNs in a controlled fashion was achieved upon lowing the solution pH. The electrostatic repulsion of positively-charged tertiary ammonium of PDMAEMAs in acidic environments induced the stretching out of polymer brushes on MSN surfaces, thus opening the gates to allow cargo diffusion out of the mesopores of MSNs.

## 1. Introduction

Enzymes/proteins are naturally-occurring, environmentally benign, and non-toxic biocatalysts [1,2,3,4]. Enzymatic polymerization possesses great potential in the production of materials for food processing and biomedical applications [5,6,7]. Nowadays, many researchers have devoted themselves to developing enzyme/protein-based green catalytic reactions for organic catalysis or (bio)polymer synthesis [8,9,10,11]. Oxidoreductases, transferases, and hydrolases are three major enzyme classes that have been successfully employed for polymer synthesis. Although oxidoreductase-catalyzed free radical polymerization has been developed for two and half decades [8,9,12], atom transfer radical polymerization (ATRP) by oxidoreductases—including horseradish peroxidase (HRP), catalase, and laccase—has emerged only within the last five years [13,14,15,16,17,18,19,20,21]. These enzymatic catalytic polymerization systems effectively avoid the use of toxic transition metal complexes, but naturally-occurring enzyme catalysts also possess some drawbacks in catalysis, such as high molecular weight and high sensitivity to reaction conditions [16,20]. Recently, naturally-occurring hematin-based peroxidase mimetics have been developed to replace natural enzymes, which showed improved performance in the construction of well-defined (bio)polymers [16,17]. Among them, deuterohemin-β-Ala-His-Thr-Val-Glu-Lys (DhHP-6) is a unique type of peroxidase mimetic with well-defined structure and low molecular weight (*M*_w_ = 1229 Da), which is easy to synthesize with renewable resources [22,23]. DhHP-6 could also serve as an ATRP catalyst to expand the monomer’s range and increase catalytic tolerance to pH variation [19].

On the other hand, much attention has been paid to mesoporous silica nanoparticles (MSNs), which are always attractive candidates for controlled drug release, biosensing, gene transfection, etc. [24,25,26,27,28], owing to their high loading capacity, good stability, and biocompatibility. A series of controlled cargo delivery systems based on MSNs have been developed through covalent or noncovalent decoration by small molecules, supramolecules, or (bio)polymers [24,25,29,30,31,32,33,34,35,36,37,38]. Poly(*N*,*N*-dimethylaminoethyl methacrylate) (PDMAEMA) containing pendant tertiary amines is a pH- and temperature-responsive polymer [39,40,41,42,43], usually used as cationic antibacterial surfaces and nonviral gene delivery carriers for medical and biomedical applications [44,45,46,47,48,49]. PDMAEMA was grafted onto the surfaces of various materials by ATRP [45,48], offering thermal- and pH-responsive nanovalves or nonviral gene vectors.

Herein, we first decorate the surface of MSNs with PDMAEMA polymer brushes by a biocompatible DhHP-6-catalyzed surface-initiated ATRP (SI-ATRP) approach in a facile and highly efficient manner that avoids the use of toxic transition metal complexes and increases catalytic performance. Rhodamine 6G (Rh6G) cargo is used as a model drug loading in the mesopores of PDMAEMA brushes-MSN hybrid materials, and its controlled release in response to pH changes is further demonstrated. The green catalysis-mediated biocompatible organic–inorganic hybrid nanomaterials hold great potential in nanomedicine and cancer therapy, and hopefully this environment-benign development will contribute to “green chemistry” for maintaining a sustainable society.

## 2. Materials and Methods

### 2.1. Materials

*N,N*-Dimethylaminoethyl methacrylate (DMAEMA) was purchased from Sigma-Aldrich (Shanghai, China), and inhibitors were removed through an alumina column before use. 2-Bromoisobutyryl bromide (BIBB) was purchased from Sigma-Aldrich and used as received. Hydroxyl-terminated MSN (MSN-OH) material was prepared according to our previously reported procedure [50]. DhHP-6 was obtained as a gift sample from College of Life Sciences, Jilin University (Changchun, China).

### 2.2. Measurements

Fourier transform infrared spectra (FT-IR) were recorded on a Shimadzu FTIR 8400S spectrometer (Kyoto, Japan). Thermogravimetric analysis (TGA) data were obtained on a TA Q500 instrument (New Castle, DE, USA). X-ray photoelectron spectroscopy (XPS) was recorded on a Thermo Scientific ESCALAB 250 spectrometer (Waltham, MA, USA). Elemental analysis was performed on a Vario EL cube elementar (Hanau, Germany). Scanning electron microscopy (SEM) and transmission electron microscopy (TEM) images were obtained on a HITACHI SU4800 instrument (Kyoto, Japan) and JEM-2100F (with an accelerating voltage of 200 kV, Kyoto, Japan), respectively. Powder X-ray diffraction (XRD) was carried out on a PANalytical B.V. Empyrean powder diffractometer (Almelo, Holland). Hydrodynamic diameter and Zeta-potentials were measured on Zetasizer Nano ZS90 (Malvern, UK), where nanoparticles were dispersed in deionized water. N_2_ adsorption–desorption isotherms were measured on an ASAP 2020 micromeritics porosimeter (Norcross, GA, USA). UV–Vis spectra were recorded on a Shimadzu UV-2550 spectrophotometer (Kyoto, Japan).

### 2.3. Methods

#### 2.3.1. ATRP-Initiator Immobilization (Synthesis of MSN-Br)

The ATRP initiator-anchored MSN (MSN-Br) was synthesized via the reaction of the hydroxy groups of MSN-OH with BIBB in the presence of triethylamine (TEA). MSN-OH (0.5 g), triethylamine (TEA, 6.8 mL), and freshly-dried toluene (150 mL) were added into a 250 mL round-bottomed flask. After ultrasonically dispersed for 1 h, the mixture was cooled in an ice-water bath, followed by the dropwise addition of BIBB (3 mL). After stirring at 0 °C for 2 h, the mixture was allowed to warm up to room temperature and stirred overnight. Crude product was isolated by centrifugation (12,000 rpm, 30 min) and then washed with chloroform (50 mL × 3), ethanol (50 mL × 1), and distilled water (50 mL × 3), respectively. The resulting MSN-Br was then dried overnight under vacuum at room temperature.

#### 2.3.2. PDMAEMA Growth on MSNs by DhHP-6-Catalyzed MSN-Br SI-ATRP of DMAEMA

MSN-Br (100 mg) was dispersed in a mixed solvent of DMF/H_2_O (5 mL, 2/3 in *v/v*) in a 10 mL round-bottomed flask, and ultrasonically dispersed for 1 h. DhHP-6 (3 mg, 0.0024 mmol) was added in the above reaction mixture. The flask was maintained at 45 °C in an oil bath, and nitrogen was bubbled through the mixture for 0.5 h. After the addition of l-ascorbic acid (0.2 mL, 2.2 mM in water), the system was sealed. DMAEMA (0.5 mL, 2.96 mmol) was then added by purging into the reaction system to start the reaction, which was then kept for 24 h under stirring. The product was separated by centrifugation (13,000 rpm, 30 min), then washed with ethanol (50 mL) and distilled water (50 mL × 3). The PDMAEMA-grafted MSN (MSN-PDMAEMA) hybrid material was obtained after drying under vacuum at room temperature.

#### 2.3.3. Rh6G Loading

MSN–PDMAEMA (50 mg) nanoparticles were dispersed in PBS of pH 2.0 (20 mL) by ultrasonication, followed by the addition of Rh6G (30 mg, 0.031 mmol). The resulting mixture was stirred at room temperature for 24 h to achieve the loading of Rh6G in the mesopores of MSNs. The product (Rh6G@MSN-PDMAEMA) was centrifuged and washed with distilled water to remove free and surface-absorbed Rh6G.

#### 2.3.4. Rh6G Release from Rh6G@MSN-PDMAEMA

Rh6G@MSN-PDMAEMA material (1.5 mg) was dispersed in 1 mL PBS buffer and then transferred into a dialysis bag (cut-off *M*_W_ = 8000−14,000 Da). The dialysis bag was immersed into different release media (9 mL PBS of pH 5.0, pH 7.4, and phosphoric acid buffer of pH 2.0), and stirred at room temperature. At certain time intervals, 3 mL of release solution was withdrawn and replenished with 3 mL of fresh buffer. The cumulative release of Rh6G was determined by UV–Vis spectroscopy at 526 nm. The calibration curves for the rhodamine 6G in different PBS buffers are provided in Figure S1 (Supplementary Material).

## 3. Results and Discussion

### 3.1. ATRP-Initiator Immobilization

**Scheme 1:**
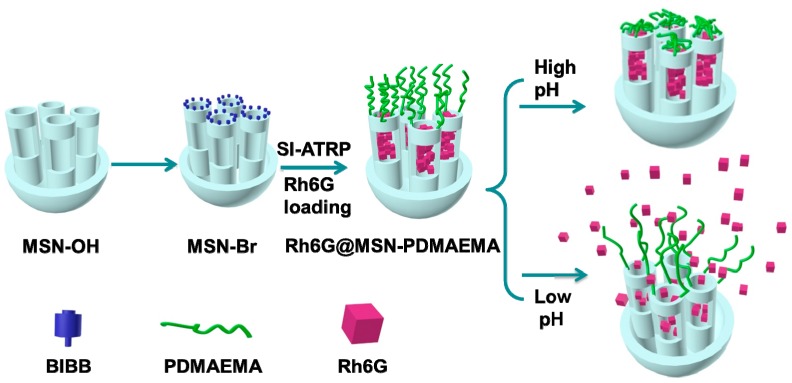
Schematic representation of the preparation process of Rh6G-loaded PDMAEMA-grafted MSN (Rh6G@MSN-PDMAEMA) via surface initiated atom transfer radical polymerization (SI-ATRP) and its pH-responsive cargo release. BIBB: 2-bromoisobutyryl bromide; MSN: mesoporous silica nanoparticles; MSN-Br: 2-bromoester-terminated MSN; MSN-OH: hydroxyl-terminated MSN; PDMAEMA: poly(*N*,*N*-dimethylaminoethyl methacrylate; Rh6G: rhodamine 6G.

**Figure 1:**
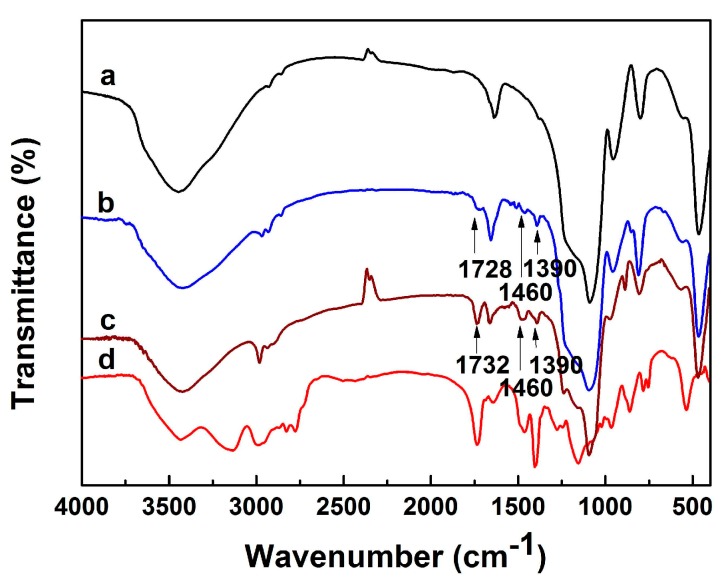
Fourier transform-infrared (FT-IR) spectra of (**a**) MSN-OH; (**b**) MSN-Br; (**c**) MSN-PDMAEMA; and (**d**) PDMAEMA.

**Figure 2:**
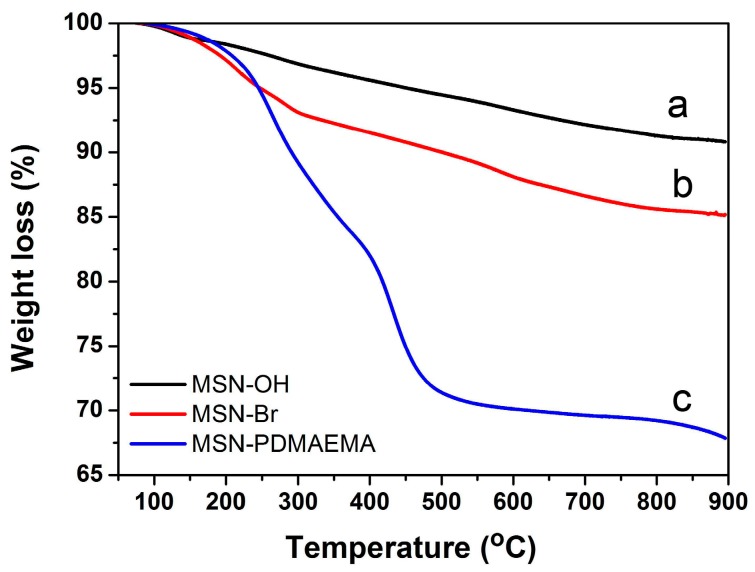
Thermogravimetric analysis (TGA) of (**a**) MSN-OH (weight loss above 100 °C was 9.1%); (**b**) MSN-Br (weight loss above 100 °C was 14.9%); and (**c**) MSN-PDMAEMA (weight loss above 100 °C was 30.7%).

In order to introduce PDMAEMA on the surface of MSNs by SI-ATRP, the hydroxyl groups of MSN-OH were converted to 2-bromoester-terminated MSN (MSN-Br) (Scheme 1). Figure 1 shows the FT-IR spectra of MSN-OH and MSN-Br. In the FT-IR spectrum of MSN-Br, the appearance of a peak at 1728 cm^−1^—attributed to the carbonyl (C=O) stretching vibrations of the ester group of the initiator—confirmed that alkyl halide was anchored to the surface of the MSNs. The increased elemental contents of C and H in MSN-Br also suggested that the ATRP initiator was introduced onto the silica surface. As MSNs are thermally-stable materials, TGA was used to analyze the weight loss of these silica-based materials (Figure 2). Weight loss of MSNs at temperatures above 100 °C was attributed to the loss of functional groups on the surface. Except for the weight loss of MSN-OH, the weight loss of MSN-Br is about 5.8%, which is also consistent with the elemental analysis results (Table 1). We can estimate that ca. 0.38 mmol·g^−1^ of alkyl halide was anchored onto MSN-OH.

### 3.2. DhHP-6 Catalyzed Synthesis of MSN-PDMAEMA and Its Characterization

In order to expand the range of DhHP-6-catalyzed polymerizable monomers and to explore the potential application of this kind of biocatalyst, PDMAEMA was grafted from the initiator-anchored surface of MSN-OH for the first time under ATRP mechanism. As we demonstrated earlier, DhHP-6 could tolerate pH in the range of 3.0–11.0; herein we chose distilled water as the reaction medium for SI-ATRP.

MSN-PDMAEMA sample was first ascertained by FT-IR spectra (Figure 1). The appearance of a peak at 1732 cm^−1^, attributed to the C=O stretching vibration of the ester groups of PDMAEMA, and the ratio of ethyl bending (1460 cm^−^^1^) to methyl bending (1390 cm^−^^1^) peak areas increases obviously compared to MSN-Br, jointly indicating that the polymer was grafted on the surface of MSNs. Compared to that of MSN-Br, the increased 15.8% weight loss of MSN-PDMAEMA (Figure 2) in TGA experiments also suggested that PDMAEMA was grafted onto the MSN surface, corresponding well with the results of elemental analysis (Table 1).

**Figure 3:**
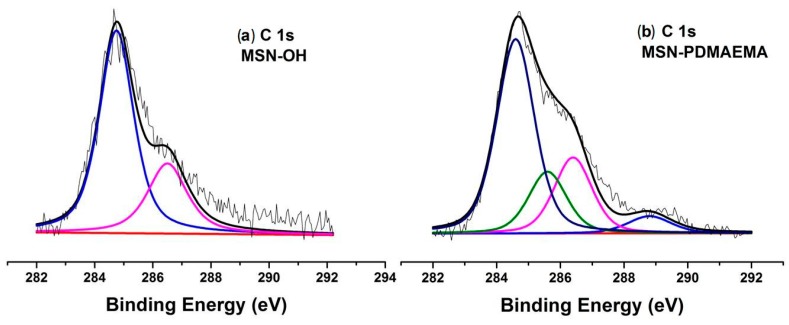
X-ray photoelectron spectroscopy (XPS) C 1s spectra of (**a**) MSN-OH (BE of 284.6 and 286.4 eV, attributed to C–H and C–O); (**b**) MSN-PDMAEMA (BE of 284.6, 285.2, 286.4, and 288.8 eV, attributed to C–H, C–N, C–O, and O=C–O).

In order to further confirm that PDMAEMA was successfully grafted from MSN-Br, XPS was employed. As shown in Figure 3b, four curve-fitted peaks with binding energy (BE) of ca. 284.6, 285.2, 286.4, and 288.8 eV can be attributed to the C–H, C–N, C–O, and O=C–O components of PDMAEMA, respectively.

**Figure 4:**
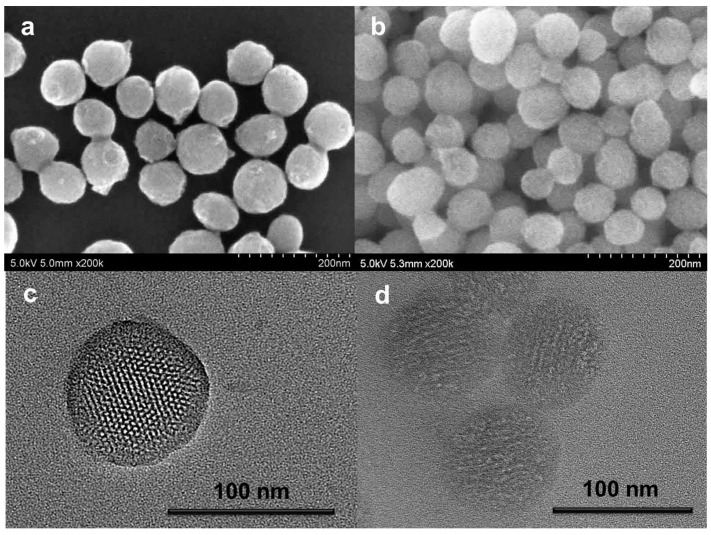
SEM images of (**a**) MSN-OH and (**b**) MSN-PDMAEMA; Magnified TEM images of (**c**) MSN-OH and (**d**) MSN-PDMAEMA.

SEM and TEM were used to demonstrate the particle morphology of MSN-OH and MSN-PDMAEMA (Figure 4). MSNs showed uniform particle size, before (MSN-OH, 79.5 ± 15.5 nm) and after (MSN-PDMAEMA, 84.6 ± 7.1 nm) DhHP-6-catalyzed SI-ATRP. A rough surface was observed in the SEM image of MSN-PDMAEMA. TEM images further revealed the existence of the ordered pore structure of MSN-OH. After PDMAEMA immobilization, the mesopores of MSNs became blurry, which is visual evidence of polymer grafting onto the surface of the MSN-OH material (Figure 4 and Figure S2).

The microcrystalline structure of MSNs could also be verified by small-angle powder XRD. MSN-OH has a similar XRD pattern to standard microporous silica (Figure S3). After PDMAEMA grafting on the MSNs surface, the intensity of diffraction peaks reduced correspondingly, in accordance with the results reported before [35,46,51,52]. Together with the TEM results, these indicate that the materials were surface-functionalized with PDMAEMA polymer brushes.

**Figure 5:**
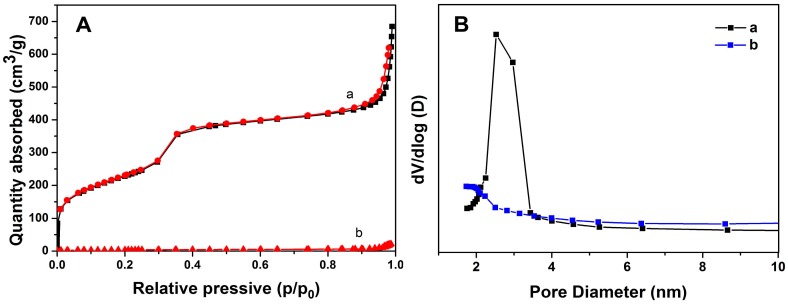
(**A**) Brunauer–Emmett–Teller (BET) nitrogen sorption isotherms and (**B**) BJH pore size distribution plots of (**a**) MSN-OH and (**b**) MSN-PDMAEMA.

The typical type IV isotherm of MSN-OH in Brunauer–Emmett–Teller (BET) nitrogen sorption isotherms confirms its mesoporous structure. The BET surface area of MSN-OH is 829 m^2^·g^−1^ (Figure 5A), and the pore diameter is about 2.4 nm (Figure 5B). In contrast, the N_2_ adsorption–desorption isotherm of the PDMAEMA grafted nanoparticle, MSN-PDMAEMA, reveals the characteristics of nonporous materials [28]. The BET surface area of MSN-PDMAEMA was decreased to 337 m^2^·g^−1^, and the pore diameter distribution and volume were also obviously changed (Table 2). These may be attributed to the fact that polymer brushes on the MSN surface avoided N_2_ penetration, which also indicated that the DhHP-6-catalyzed SI-ATRP on the MSNs surface was successful.

### 3.3. Rh6G Release from Rh6G@MSN-PDMAEMA in Solutions of Different pH Values

**Figure 6:**
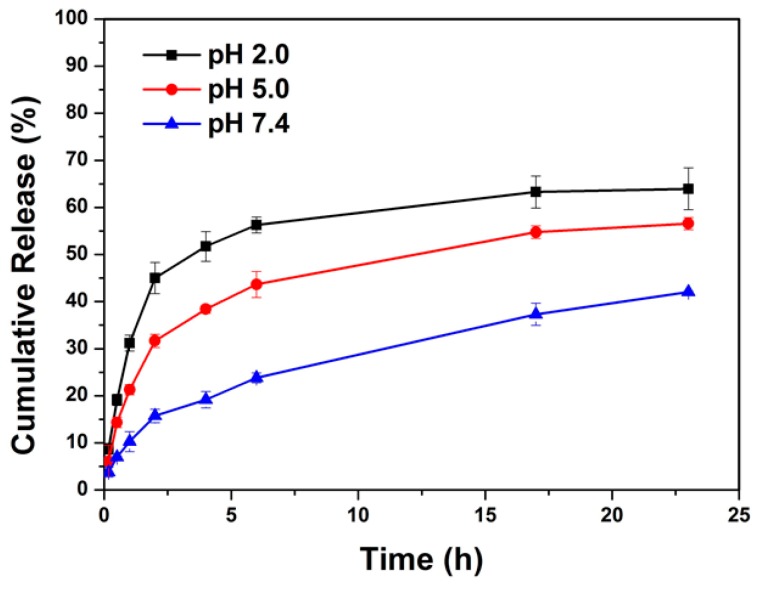
Cargo release profiles of Rh6G@MSN-PDMAEMA in different PBS buffers.

Tertiary amine groups of the PDMAEMA side chains could be protonated at lower pH, resulting in the stretching out of polymer brushes on MSNs due to strong electrostatic repulsion, thus opening the gates on MSNs to allow cargo release [48,53,54]. At neutral pH, the deprotonation of PDMAEMA polymer chains makes them stay on the MSNs surface in a shrunken/collapsed state to reduce surface tension; therefore, MSNs pores will be blocked by the polymer layer and prevent cargo release. In a typical proof-of-concept study, Rh6G was loaded into MSN-PDMAEMA to result in Rh6G@MSN-PDMAEMA. The conformational states of PDMAEMA on MSNs change in different pH conditions, as shown in Scheme S1. Cargo release was monitored in buffer solutions of different pH values (Figure 6). At pH 2.0, fast release was observed; there was about 56% cumulative release within 8 h. At pH 5.0, the release reached a plateau in 30 h, and the cumulative release amount of Rh6G was 57%. At pH 7.4, the release speed was quite low and the final release content was ca. 42%. Therefore, we can conclude that MSN-PDMAEMA constructed from DhHP-6-catalyzed SI-ATRP also exhibited a pH-dependent cargo release property. Apparent Zeta-potential dependency of MSNs toward pH values of different PBS buffers was then investigated (Figure S4). The Zeta-potential of nanoparticles decreased with increasing pH values of the MSN suspensions. The critical electrostatic equilibrium pH of Rh6G@MSN-PDEAEMA was determined to be about 7.0, which means that the MSN-grafted PDEAEMA should be deprotonated at the pH value of blood circulation (pH 7.4). Based on this observation, this DhHP-6 catalyzed SI-ATRP of PDEAEMA could be used as pH-dependent gatekeeper on the surface of MSNs for pH-controlled sustained drug release. The final loading and delivery properties of this system are expected to be fine-tuned by controlling the grafting degree of polymer brushes on the MSN surface and adjusting the polymer components, which is the on-going research in our laboratory.

## 4. Conclusions

MSNs were successfully modified with PDMAEMA polymer brushes by DhHP-6-catalyzed SI-ATRP, a green biocatalytic approach without toxic residues in polymers after reaction. Elemental analysis, TGA, XPS, TEM, SEM, and Zeta-potential measurements confirmed that PDMAEMA brushes were immobilized on the surface of MSNs. PDMAEMA-modified MSNs exhibited a typical pH-responsive cargo release behavior and showed potential applications in controlled drug release and gene delivery. This biocompatible DhHP-6 catalyzed ATRP on MSNs surface may open up a new avenue on the construction of more useful controlled drug release systems gated by polymeric materials. More biocatalysts should be tested and used in this research field for the fast development of drug delivery and controlled release.

## Figures and Tables

**Table 1:** Elemental analysis of MSN-based materials.

Materials	C (%)	H (%)	N (%)
MSN-OH	4.67	2.07	0.42
MSN-Br	10.19	2.81	0.22
MSN-PDMAEMA	15.61	3.63	1.62

**Table 2:** Mesopore parameters of MSN-OH and MSN-PDMAEMA.

Sample	BET surface area/m^2^·g^−1^	Volume of pores/cm^3^·g^−1^
MSN-OH	829.32	0.98
MSN-PDMAEMA	337.22	0.69

## Supplementary Materials

Supplementary Materials can be found at www.mdpi.com/2073-4360/8/8/277/s1.
