# Supplementary material for: Surface Immobilization of pH-Responsive Polymer Brushes on Mesoporous Silica Nanoparticles by Enzyme Mimetic Catalytic ATRP for Controlled Cargo Release
Source: Polymers (Basel). 2016 Aug 2;8(8):277. doi: 10.3390/polym8080277 (PMC6432388; doi:10.3390/polym8080277)
Supplement: Supplementary file 1 [file polymers-08-00277-s001.pdf]

Supplementary Materials: Surface Immobilization of pH-Responsive Polymer Brushes on Mesoporous Silica Nanoparticles by Enzyme Mimetic Catalytic ATRP for Controlled Cargo Release

Hang Zhou, Xin Wang, Jun Tang and Ying-Wei Yang

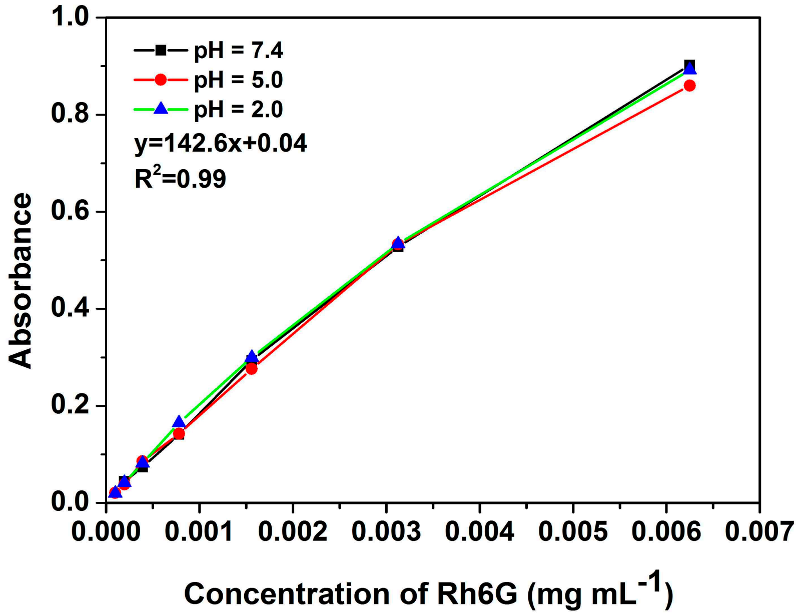

Figure S1. Calibration curves for rhodamine 6G in different PBS buffers.

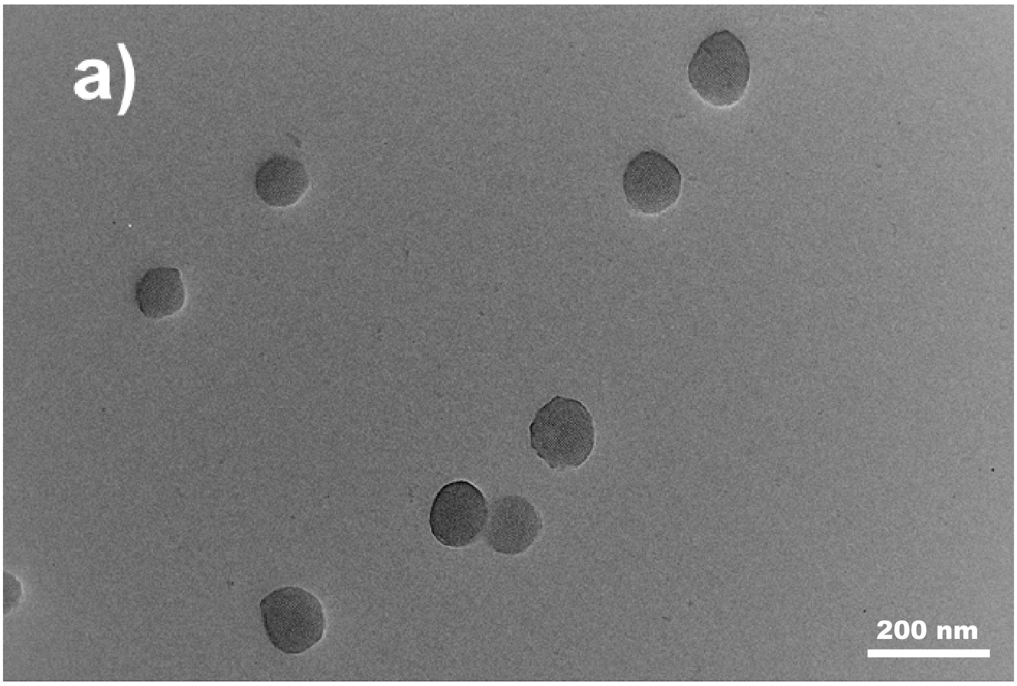

Figure S2. *Cont.*

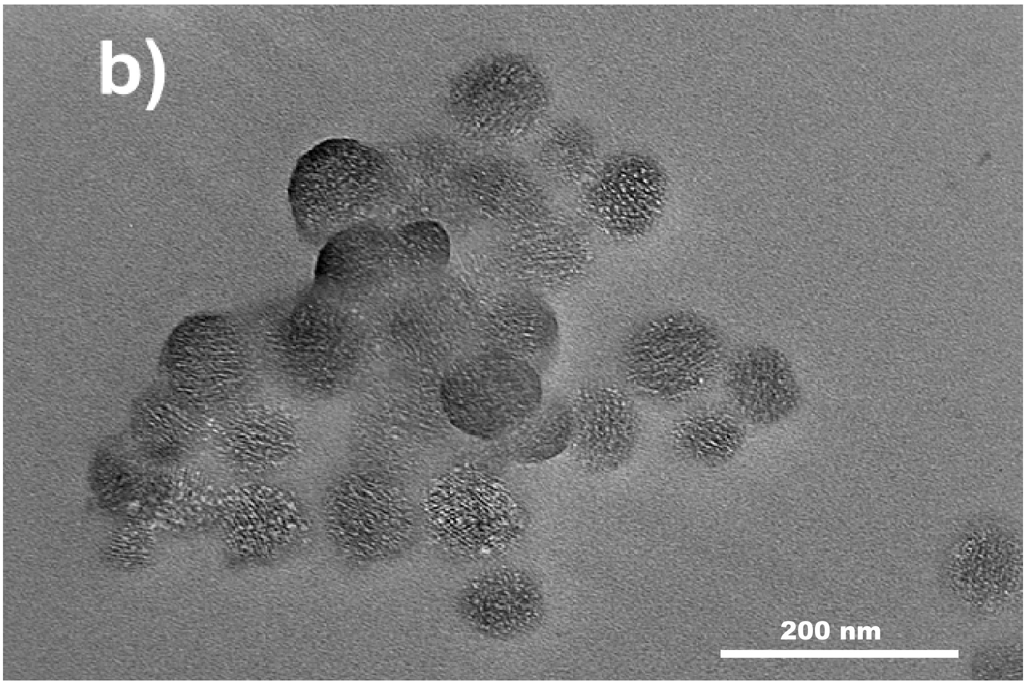

Figure S2. TEM images of (**a**) MSN-OH and (**b**) MSN-PDMAEMA.

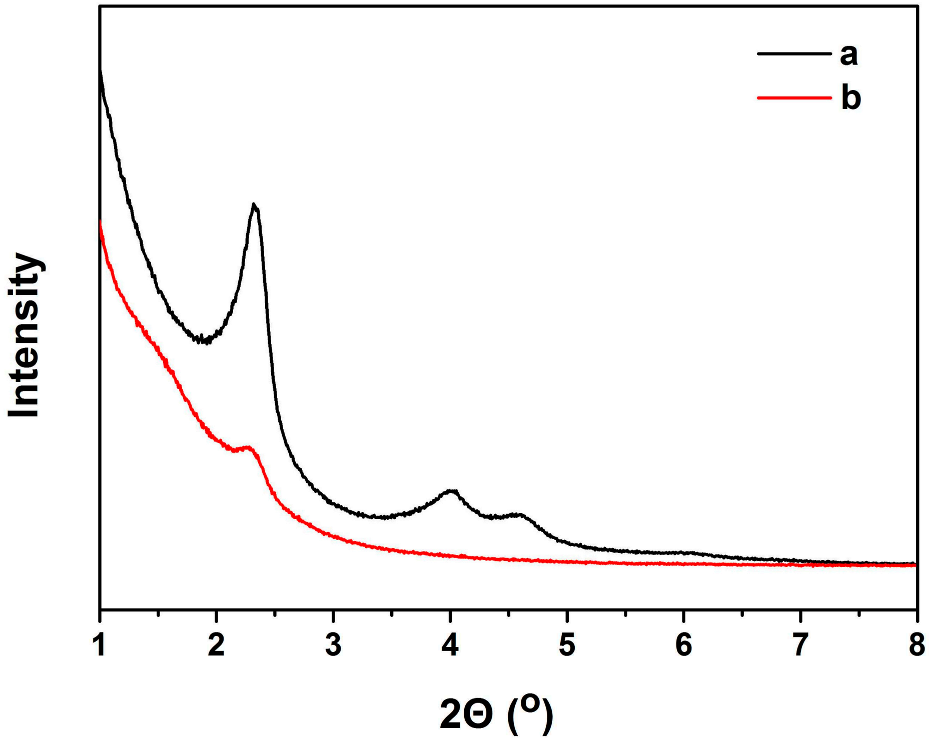

Figure S3. The small-angle powder XRD of (**a**) MSN-OH and (**b**) MSN-PDMAEMA.

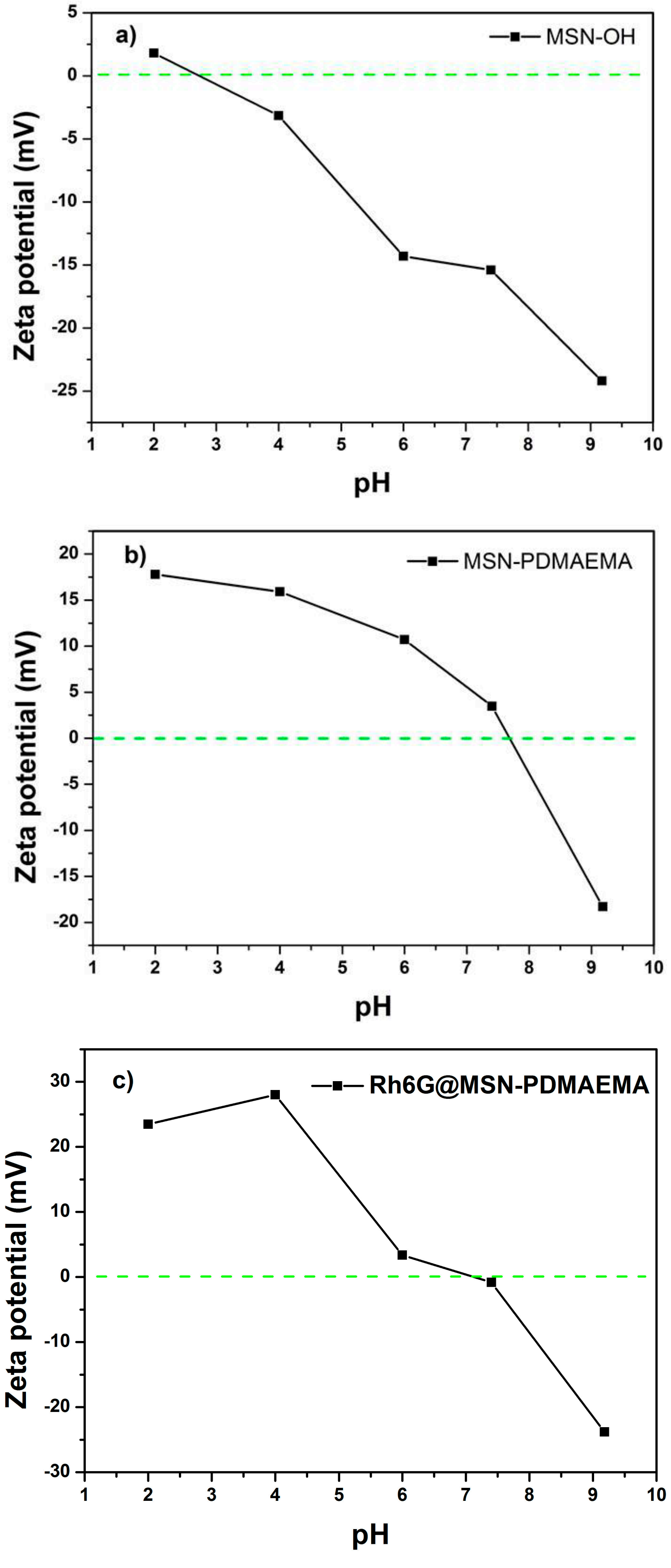

Figure S4. pH-Dependency of the apparent Zeta-potential values of MSNs in different PBS buffers: (**a**) MSN-OH; (**b**) MSN-PDMAEMA; and (**c**) Rh6G@MSN-PDMAEMA.

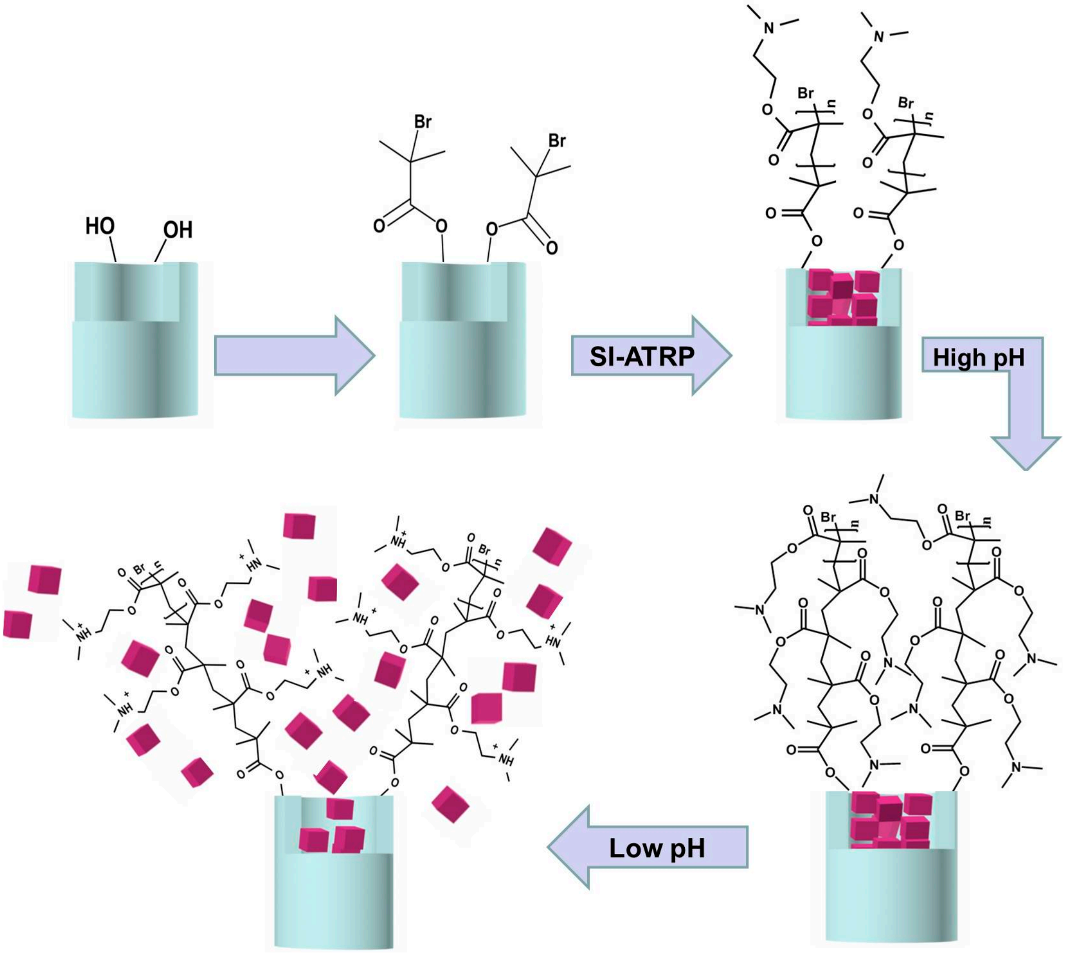

Scheme S1. Schematic representation of the preparation of Rh6G@MSN-PDMAEMA via SI-ATRP and the operation of the switching of polymer brushes on MSNs by pH changes.

© 2016 by the authors; licensee MDPI, Basel, Switzerland. This article is an open access article distributed under the terms and conditions of the Creative Commons Attribution (CC-BY) license (http://creativecommons.org/licenses/by/4.0/).
